# Galactose Enhances Chondrogenic Differentiation of ATDC5 and Cartilage Matrix Formation by Chondrocytes

**DOI:** 10.3389/fmolb.2022.850778

**Published:** 2022-05-09

**Authors:** Zhongrun Yuan, Sa Liu, Wenjing Song, Ying Liu, Gangyuan Bi, Renjian Xie, Li Ren

**Affiliations:** ^1^ School of Materials Science and Engineering, South China University of Technology, Guangzhou, China; ^2^ National Engineering Research Center for Tissue Restoration and Reconstruction, South China University of Technology, Guangzhou, China; ^3^ Guangdong Key Laboratory of Biomedical Engineering, South China University of Technology, Guangzhou, China; ^4^ School of Biomedical Sciences and Engineering, South China University of Technology, Guangzhou, China; ^5^ Key Laboratory of Prevention and Treatment of Cardiovascular and Cerebrovascular Diseases, Ministry of Education, Gannan Medical University, Ganzhou, China; ^6^ School of Medical Information Engineering, Gannan Medical University, Ganzhou, China; ^7^ Jiangxi Key Laboratory of Medical Tissue Engineering Materials and Biofabrication, Gannan Medical University, Ganzhou, China

**Keywords:** galactose, chondrogenesis, cartilage matrix formation, cartilage repair, Leloir pathway, glycosaminoglycan

## Abstract

Galactose, an important carbohydrate nutrient, is involved in several types of cellular metabolism, participating in physiological activities such as glycosaminoglycan (GAG) synthesis, glycosylation, and intercellular recognition. The regulatory effects of galactose on osteoarthritis have attracted increased attention. In this study, *in vitro* cell models of ATDC5 and chondrocytes were prepared and cultured with different concentrations of galactose to evaluate its capacity on chondrogenesis and cartilage matrix formation. The cell proliferation assay demonstrated that galactose was nontoxic to both ATDC5 cells and chondrocytes. RT-PCR and immunofluorescence staining indicated that the gene expressions of cartilage matrix type II collagen and aggrecan were significantly upregulated with increasing galactose concentration and the expression and accumulation of the extracellular matrix (ECM) protein. Overall, these results indicated that a galactose concentration below 8 mM exhibited the best effect on promoting chondrogenesis, which entitles galactose as having considerable potential for cartilage repair and regeneration.

## 1 Introduction

Cartilage tissue is frequently subjected to external stresses, strains, and loads on a daily basis (Fan and Waldman, 2010). Nutrients from the synovial fluid freely and inefficiently diffuse through the pores in the cartilage to reach chondrocytes (Park et al., 2007; Haskó et al., 2018; Kaplan et al., 2019). In addition, due to the avascular nature of cartilage, its ability to self-repair and regenerate is limited once damaged or injured (Schminke and Miosge, 2014). Therefore, the methods used to treat cartilage lesions, such as osteoarthritis (OA), and induce regeneration are important. Many saccharide molecules, such as chondroitin sulfate and glucosamine, have been shown to be beneficial for cartilage regeneration and even have anti-inflammatory effects (Varghese et al., 2007), but the cost of industrial extraction of natural chondroitin sulfate remains high and glucosamine is currently available in some countries as a common dietary supplement or a drug that protects cartilage (Handl et al., 2007; van de Water et al., 2017). However, some clinical data showed no obvious evidence of improvement following glucosamine supplementation in patients with chronic knee joint diseases (Durmus et al., 2012; Korkmaz et al., 2013; Kwoh et al., 2014), and thus, the effect is inconsistent and controversial. Hence, the search for other more effective compounds for cartilage repair is still a major topic of research.

In chondrocytes, the conversion and utilization of galactose, an upstream anabolic molecule, into the metabolically more useful glucose-1-phosphate are accomplished by four enzymes that constitute the Leloir pathway (Holden et al., 2003). Chondrocytes can secrete a range of proteoglycan precursors *via* the Leloir pathway in the Golgi apparatus, which contributes to the production of glycosaminoglycans (GAGs) such as chondroitin sulfate and keratan sulfate and glycosylation between lipid molecules and proteins and proteins and carbohydrates. (Knudson and Knudson, 2001; Pavão et al., 2006). Furthermore, galactose acts as the main component monosaccharide of the linking bridge between protein–glycan molecular chains. Long-chain galactosyltransferases (β1,4-GalT-I isomers) at the cell surface can bind with oligosaccharide substrates or ligands of N-acetyl glucosaminoglucose in the extracellular matrix (ECM), that is, galactose, which acts as an intercellular and cellular-matrix adhesive in the interaction of individual cells, thus triggering cellular responses (Liu et al., 2012; Wen et al., 2014). However, the potential of galactose to regulate the chondrogenic phenotype has rarely been exploited.

Chondrocytes, the only embedded cells in cartilage, are widely utilized in cartilage-related *in vitro* studies. Controlling the cell phenotype in the cartilage matrix is essential in addressing the clinical challenge of OA. However, chondrocytes are relatively difficult to isolate and prone to dedifferentiation when cultured *in vitro*, which is expensive (Gosset et al., 2008). However, encouragingly, the ATDC5 cell line derived from mouse embryonic carcinoma by Atsumi in 1990 has been shown to retain the properties of cartilage progenitor cells, exhibits more efficient cartilage differentiation, and is more stable than other cell lines *in vitro* (Atsumi et al., 1990; Yao and Wang, 2013; Yao et al., 2020). In addition, the ATDC5 cell line proliferated rapidly and is easy to expand and preserve *in vitro*; therefore, this cell line is also widely used for chondrogenic differentiation studies (Caron et al., 2013; Caron et al., 2021).

**SCHEME 1:**
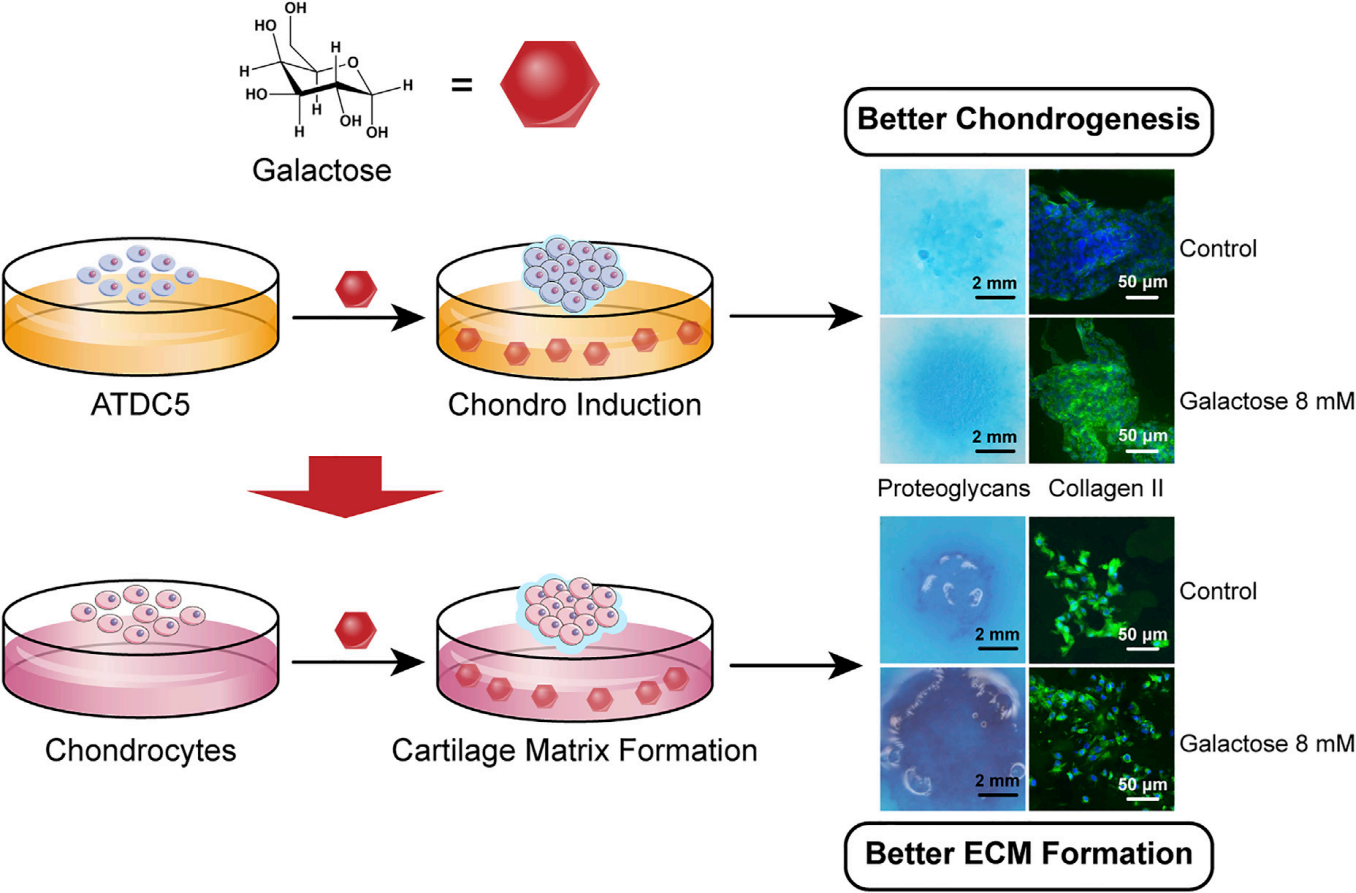
Galactose promotes chondrogenic differentiation of ATDC5 and cartilage matrix formation of chondrocytes.

To date, few studies have investigated the effects of galactose on chondrogenesis. Given these findings, as shown in Scheme 1, this study aimed to establish the relationship between cellular biological functions and galactose. The cytotoxicity of galactose on ATDC5 and chondrocytes and the optimal concentration to promote chondrogenesis and cartilage matrix formation were investigated to explore a potential biomaterial for cartilage repair and regeneration.

## 2 Materials and Methods

### 2.1 ATDC5 Cell Culture and Differentiation

All cell culture reagents were acquired from Gibco^®^ by Life Technologies, unless otherwise stated. The ATDC5 cell line was purchased from Xiangf Bio (Shanghai, China). The cells were cultured and passaged in the normal medium of 1:1 mixture (v/v) of Dulbecco’s modified Eagle’s medium and Ham’s F12 medium (DMEM/F12) with 10% (v/v) fetal bovine serum (FBS). The eighth passage (P8) was used. For chondrogenic differentiation, the macromass method was applied as reported previously (Yao et al., 2017). A 10 μL ATDC5 cell suspension with 2×10^7^ cells/mL was added dropwise into the center of each well of 24-well plates (Corning, United States), incubated for 2 h, and then cultured using a chondrogenesis medium with 2, 8, 35, and 70 mM galactose (Aladdin, Shanghai, China) for 21 days. The cells in the negative control group (marked as ATDC5) were maintained using a normal medium, and the cells in the positive control group (marked as 0 mM) were maintained using a chondrogenic medium, that is, DMEM/F12 was supplemented with 10% FBS, 1 mM sodium pyruvate, 40 μg/ml L-proline (Sigma, United States), 1% (v/v) ITS-G, 50 μg/mL l-ascorbic acid (Sigma), 100 nM dexamethasone (MCE, United States), and 1% (v/v) penicillin/streptomycin. The medium was changed every 2 days.

### 2.2 Isolation and Culture of Chondrocytes

Rabbit articular chondrocytes were isolated as described in the literature with slight modification (Pei et al., 2009; Gong et al., 2010). In brief, rabbit chondrocytes were harvested from the articular cartilage of five 3-week-old white rabbits (Huadong Xinhua Laboratory Animal Farm, Guangzhou, China). Cartilage was extracted from the femoral condyles and the posterior surface of the patella by excision with a scalpel. Then, the cartilage tissue was minced into small fragments under aseptic conditions. Subsequently, the cartilage slices were digested in 0.1% (w/v) collagenase type II (Sigma-Aldrich, United States) for 14 h in an incubator at 37°C and 5% CO_2_. The cell suspensions were filtrated through a Falcon^®^ Cell Strainer (mesh size 40 μm). All isolated chondrocytes were suspended in DMEM with 10% (v/v) FBS, 10 mM HEPES, 0.1 mM MEM NEAA, 0.4 mM L-proline (Sigma-Aldrich), 50 mg/L L-ascorbic acid (Sigma-Aldrich), 100 U/ml penicillin, and 100 mg/L streptomycin. In this study, chondrocytes at the second passage (P2) were used.

For cartilage matrix formation, the macromass method was used was used as described previously. Briefly, 10 μl of a cell suspension of 2×10^7^ cells/mL was dropped into the center of each well of 24-well plates and incubated in the incubator for 2 h. Then, the cultures were maintained using a medium with 2, 8, 35, and 70 mM galactose (Aladdin, Shanghai, China) for 21 days. The control group was marked as 0 mM, and the medium was replaced every 2 days.

### 2.3 Cytotoxicity and the Half-Maximal Inhibitory Concentration (IC_50_) Curve

The cytotoxicity of various concentrations of galactose toward ATDC5 cells and chondrocytes was characterized on days 1, 3, 5, and 7 using Cell Counting Kit-8 (CCK-8, Dojindo, Japan) assays according to the manufacturer’s instructions. The cells were seeded in 96-well plates (Corning, United States) at a density of 2000 cells/well, ATDC5 cells were maintained in a normal medium, and both types of cells were supplemented with galactose at a range of concentrations. At the indicated time point, the cells were incubated for 4 h in a CCK-8 working solution at 37°C and 5% CO_2_. After incubation, the absorbance of the solution was measured at 450 nm using a Thermo 3,001 microplate reader (Thermo Scientific, United States). The IC_50_ curves were generated by adding different concentrations of galactose to the medium, and the cells were cultured for 72 h, followed by cytotoxicity measurements using the CCK-8 assay.

### 2.4 Alcian Blue Staining for the ECM

The cells on days 3, 7, 14, and 21 were fixed in 4% paraformaldehyde (Macklin, China) for 12 h at 4°C and stained with 1% (w/v) Alcian Blue 8GX (Solarbio, China) aqueous solution (Challa et al., 2010). The stained cells were assembled on plates, washed three times with deionized water to ensure that only specific staining remained, and photographed using a stereomicroscope (Jiangnan, China), and the relative staining level was processed using ImageJ software.

### 2.5 Quantification of GAG Production

At the specified time points, the cells were washed with PBS three times and then were frozen in liquid nitrogen and lyophilized for 12 h. The remaining cells were collected and digested with the GAG digestion solution (papain 0.0156 g, NaOH 0.2 g, EDTA 0.0731 g, sodium dihydrogen phosphate 0.39 g, and cysteine 0.0304 g in 25 ml ultrapure water) for 16 h at 60°C. The GAG content was measured using the Blyscan sGAG Assay (Biocolor, United Kingdom), and the standard curve was generated by chondroitin sulfate standard using the kit. The DNA content was quantified using Hoechst 33,258 (Biosharp, China). Calf thymus DNA (Sigma) was used to generate a standard curve (Farndale et al., 1986).

### 2.6 Quantitative Real-Time PCR Analysis

At the specified time points, total RNA was isolated using the HiPure Total RNA Kit (Magentec., China) and the concentration of RNA was determined using a Nano drop 2000 spectrophotometer (Thermo Scientific). Then, the cDNA was reverse-transcribed using a PrimeScript RT reagent kit with gDNA Eraser (TaKaRa Biotechnology, Japan) according to the manufacturer’s protocol. Finally, quantitative real-time PCR was performed using a SYBR Green System (GeneCopoeia, United States) on an RT-PCR instrument (LightCycler 96, Roche). The relative quantification of target genes was performed with normalization to glyceraldehyde 3-phosphate dehydrogenase (GAPDH), and the 2^−ΔΔCt^ method was used to calculate the gene expression. The PCR primers used in this study are listed in Supplementary Tables S1, S2.

### 2.7 Immunofluorescence Staining and Histology Staining

Induced cells were fixed in 4% paraformaldehyde for 12 h at 4°C. The cell aggregates were carefully collected from the plates and immersed in a gradient concentration of sucrose solution (10, 20, and 30% w/v) to dehydrate the frozen sections. Then, the cell aggregate samples were embedded at optimal cutting temperature (O.C.T) and cut into 6-μm-thick sections using a freezing microtome (Leica). For immunofluorescence staining, the samples in the sectioned slides were permeabilized and blocked with 0.3% Triton X-100 and 10% goat serum for 2 h at room temperature. The samples were incubated with primary antibodies against aggrecan (1:200, Affinity), collagen II (1:200, Bioss), collagen I (1:200, Bioss), and collagen X (1:200, Bioss) overnight at 4°C. The secondary antibody was diluted 1:500 (goat antirabbit IgG FITC-conjugated, goat antirabbit IgG Fluor 594-conjugated, Affinity), and the sections were incubated with the secondary antibody for 2 h at room temperature. Then, the chondrocyte mass sections were stained with H&E, safranin-O, and Masson. The images were taken using a digital pathology scanning system (3DHISTECH P250 FLASH).

### 2.8 Statistical Analysis

All data are presented as mean ± standard deviation (STDEV) for at least three repeated individual experiments. Significant differences were confirmed by analysis of variance (two-way ANOVA) for independent samples. Statistical significance was defined as *p* < 0.05.

## 3 Results

### 3.1 Effects of Galactose on ATDC5 Cell Proliferation

After treatment for 72 h, cell behavior exhibited a significant dose-dependent relationship with galactose. The IC_50_ represents the concentration of a drug that is required for 50% inhibition (Sebaugh, 2011; Swinney, 2011). The IC_50_ value of galactose for ATDC5 was approximately 99.6 mM (Supplementary Figure S1A), which is considerably higher than that of glucosamine, which was 13 mM (Yao et al., 2017). Generally, an IC_50_ value greater than 1 mM is considered to be free of any toxicity, demonstrating that galactose can be used for more promising applications.

To further elucidate the cytotoxic effect of galactose on ATDC5 cells, CCK-8 assays were carried out at various time points during 7 days of culture. During the culture, ATDC5 showed an increasing trend of proliferation with excellent growth. For ATDC5, on days 5 and 7, cell proliferation was slightly lower in the experimental groups than that in the control group (Supplementary Figure S1B), but there was no significant difference among all ATDC5 groups, indicating that no evident cytotoxicity was observed below the galactose concentration of 32 mM. Based on these results, 2, 8, 35, and 70 mM were selected for the following tests.

### 3.2 GAG Accumulation in Chondrogenesis

**FIGURE 1:**
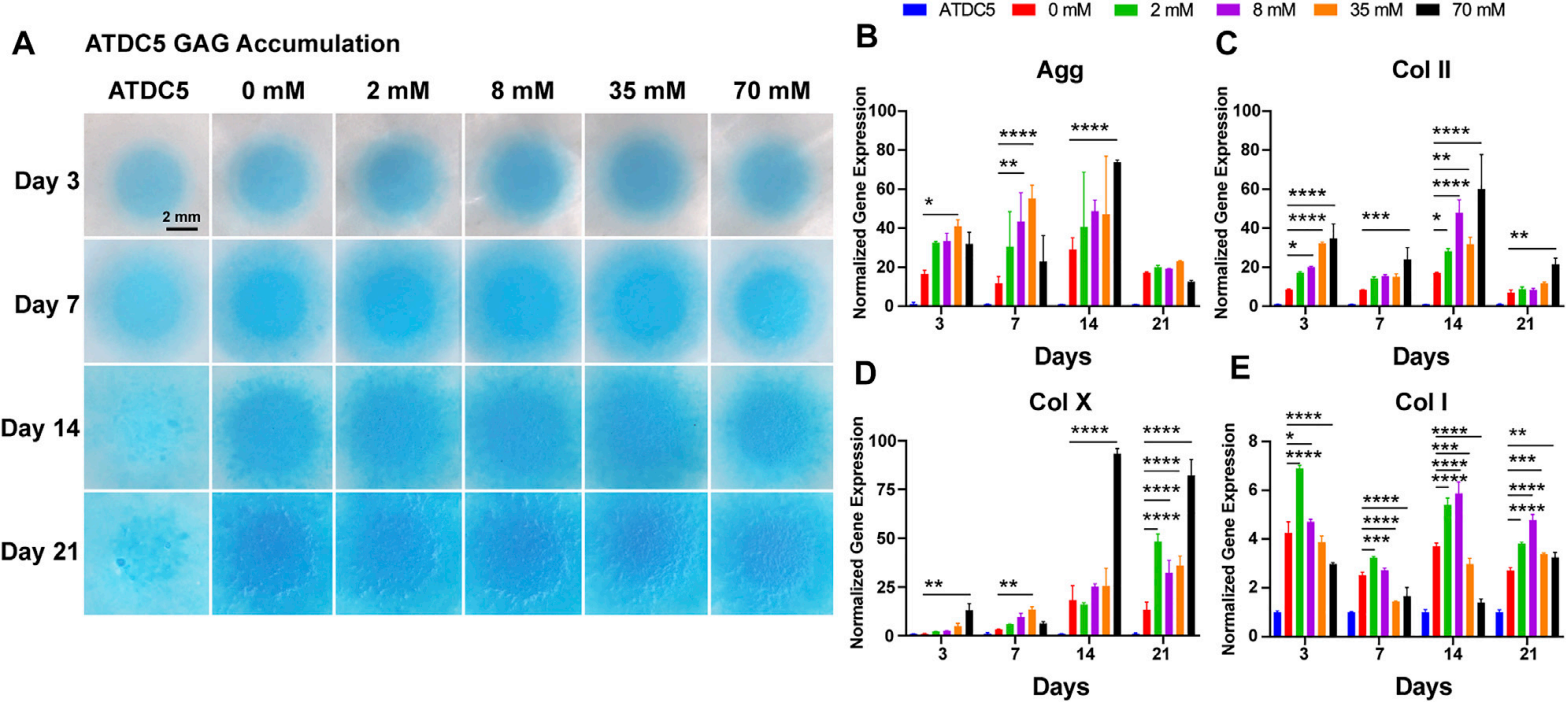
Chondrogenic phenotype of ATDC5. **(A)** GAG accumulation of ATDC5 with galactose treatment within 21 days. Scale bar = 2 mm. Gene expression analysis of **(B)** aggrecan, **(C)** collagen II, **(D)** collagen X, and **(E)** collagen I was performed on days 3, 7, 14, and 21. GAPDH was used as a reference gene. The fold change of each gene was normalized against the ATDC5 group (n = 3, *p < 0.05 vs. the 0 mM group, **p < 0.01 vs the 0 mM group, ***p < 0.001 vs the 0 mM group, and ****p < 0.0001 vs the 0 mM group).

The ECM of hyaline cartilage in knee joints, which mainly includes proteoglycans and type II collagen, has a unique physiological function and plays an important role in daily life. This entitles glycosaminoglycan (GAG) deposition as an important phenotypic parameter for chondrogenesis and chondrogenic differentiation. Alcian blue, a cationic dye, chemically binds to the acidic functional groups in GAG, so the accumulation of GAG in the deposited cartilage ECM can be directly observed by Alcian blue staining. In Figure 1A, staining in the 0 mM group (chondrogenic medium) was stronger than that in the undifferentiated group of ATDC5 (normal medium), demonstrating successful chondrogenic differentiation of ATDC5 cells. In addition, the galactose-treated groups showed more pronounced staining as the galactose concentration increased with time, suggesting increased GAG deposition in the ECM. The staining intensity of Alcian blue was quantified using ImageJ to measure the tiny difference and the quantification of the GAG/DNA content. The 8 mM group reached a peak of 1.85 ± 0.072 μg per μg DNA on day 21, significantly higher than that of the control group (Supplementary Figure S2). Therefore, galactose can enhance GAG accumulation during ATDC5 chondrogenic differentiation.

### 3.3 Chondrogenic Gene Expression

ATDC5 cells were assessed on days 3, 7, 14, and 21 for cartilage-related genes, such as aggrecan, type II collagen, type X, and type I collagen, to further define the ability of galactose to regulate the cell phenotype and promote chondrogenic differentiation. As depicted in Figure 1, the 0 mM group (positive control) showed elevated expression levels of all four genes compared to those of the undifferentiated ATDC5 (negative control), indicating that the applied differentiation method was effective. The expression levels of the hyaline cartilage–related genes aggrecan (Figure 1B) and type II collagen (Figure 1C) increased over time during the first 14 days and significantly upregulated with increasing galactose concentrations. However, the results of the type X collagen gene (a marker of cellular hypertrophy, Figure 1D) showed that the high concentrations of galactose after 14 days could lead to cell hypertrophy. In contrast, the expression of the type I collagen gene (a typical fibrocartilage marker, Figure 1E) was significantly upregulated at 2 mM for the first 3 days and peaked at 8 mM on days 14 and 21, but the expression level was much lower than that of hyaline cartilage–related genes, suggesting that galactose could effectively promote chondrogenic differentiation of ATDC5 cells.

### 3.4 Evaluation of Cartilage-Related Protein Expression *In Vitro*

**FIGURE 2:**
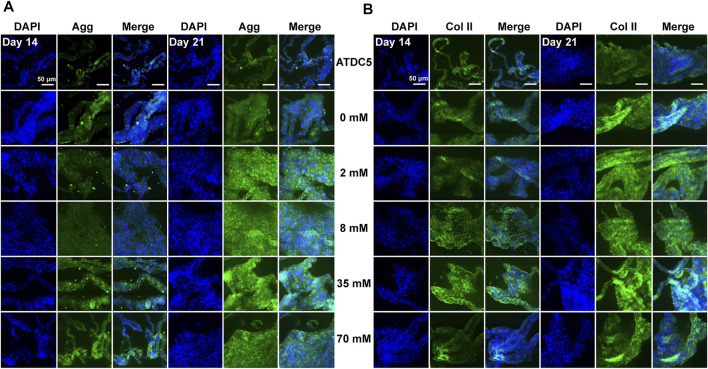
Immunofluorescence staining for the chondrogenic markers **(A)** aggrecan and **(B)** type II collagen of ATDC5. Aggrecan and type II collagen were stained green and nuclei were stained blue. Scale bar = 50 µm.

After assessment of gene expression levels, immunofluorescence staining was performed under the same conditions to detect the protein levels of aggrecan, type II, type X, and type I collagen in ATDC5 cells. Overall, the positive staining of these proteins with different intensities was observed in all experimental groups on days 14 and 21, but the cartilage-related protein expression was largely consistent with gene expression levels. For ATDC5, staining was slightly stronger on day 21 than on day 14, and the expression levels of aggrecan (Figure 2A) and type II collagen (Figure 2B) were upregulated with increasing galactose concentrations, demonstrating that galactose can successfully promote chondrogenesis. In addition, as investigated in other articles, the estimated half-life of proteoglycans is about 26 days, while that of collagen is over 100 years (Lai et al., 2013; Linka et al., 2016; Christiansen-Weber et al., 2018). Both are longer than the experiment period in this work. Although the gene expression levels of aggrecan and type II collagen were lower on day 21 than those on day 14, the experimental groups still had higher gene expression levels than those of the control group. Therefore, it is possible to infer that aggrecan and type II collagen proteins were being synthesized, and the accumulation was observed higher on day 21 than that on day 14 in the immunofluorescence staining in Figure 2. However, the expression of type X collagen (Supplementary Figure S3A) was not significantly different from that in the other experimental groups, suggesting that gene expression levels may not directly contribute to differences in protein levels. The differences among the groups treated with galactose concentrations under 8 mM and the control groups were not evident for type I collagen (Supplementary Figure S3B). Collectively, these results demonstrated that galactose at concentrations under 8 mM had a better effect on chondrogenesis.

### 3.5 Effects of Galactose on Chondrocyte Proliferation

Having shown that galactose successfully promoted chondrogenic differentiation of ATDC5 cells, we then attempted to verify the direct effect of galactose on the chondrocyte phenotype. The IC_50_ value of galactose on chondrocytes was approximately 498.5 mM (Supplementary Figure S4A), which may indicate that chondrocytes are better adapted to a high-sugar environment than ATDC5. During the 7-day culture (Supplementary Figure S4B), chondrocytes presented a similar increasing trend of proliferation as ATDC5. On days 5 and 7, cell proliferation was slightly lower in the experimental groups than that in the control group, but still higher than 80%. Galactose concentrations of 2, 8, 35, and 70 mM were selected for the following tests, consistent with those for the ATDC5.

### 3.6 GAG Accumulation in Chondrocytes

**FIGURE 3:**
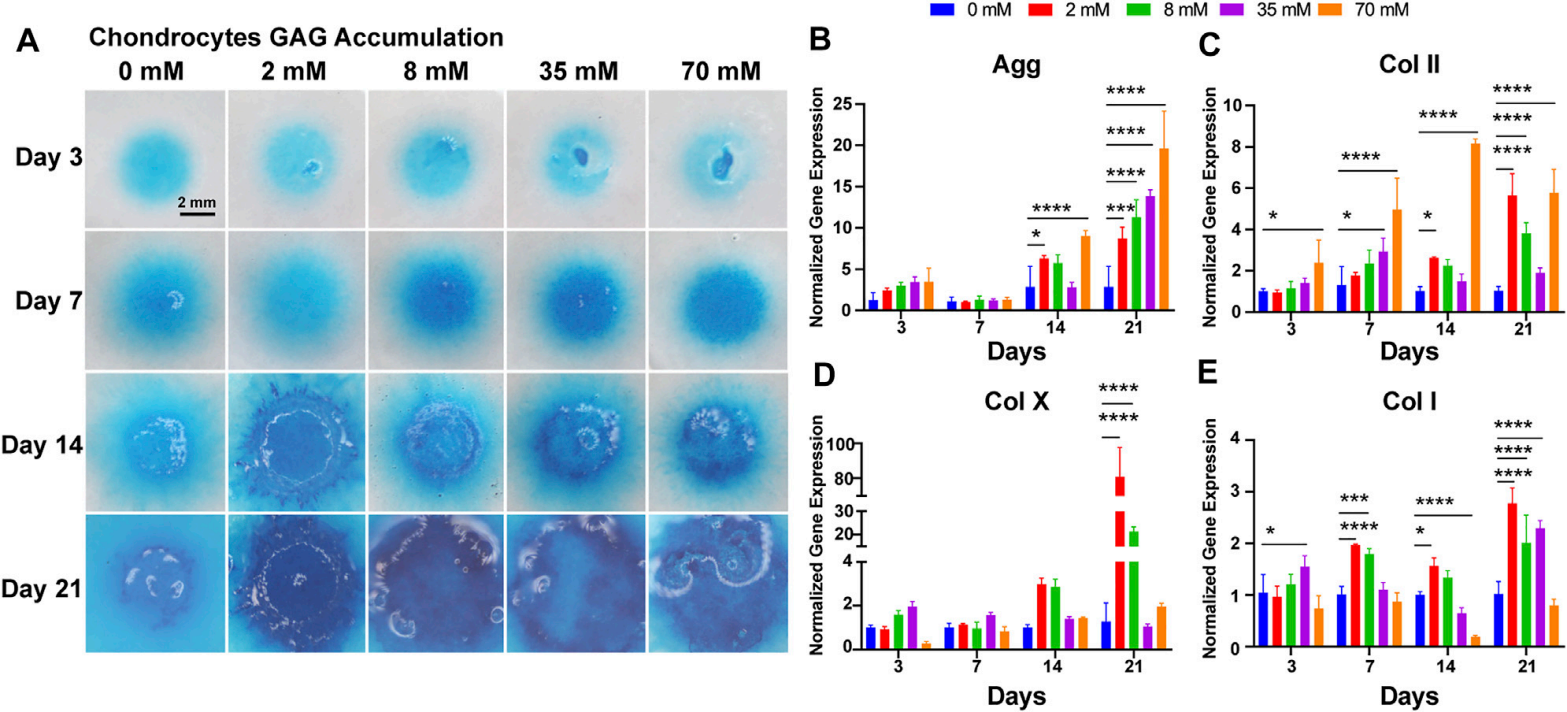
Chondrogenic phenotype of chondrocytes. **(A)** GAG accumulation of chondrocytes with galactose treatment within 21 days. Scale bar = 2 mm. Gene expression analysis of **(B)** aggrecan, **(C)** collagen II, **(D)** collagen X, and **(E)** collagen I was performed on days 3, 7, 14, and 21. GAPDH was used as a reference gene. The fold change of each gene was normalized against the 0 mM group (n = 3, *p < 0.05, **p < 0.01, ***p < 0.001, and ****p < 0.0001).

To evaluate the capacity of cartilage matrix formation by chondrocytes, cell masses were cultured at different concentrations of galactose for 21 days, followed by Alcian blue staining. All cell masses gradually became larger and thicker and proliferated outward with the extension of time. As shown in Figure 3A, the chondrocyte mass exhibited stronger staining as the galactose concentration increased with time, directly indicating that the ability of chondrocytes to generate GAG was robustly promoted by galactose stimulation. Quantitative measurements also indicated that GAG accumulation was significantly promoted with the addition of galactose, peaking on day 21 at 8 mM (35.63 ± 1.82 μg per μg DNA, Supplementary Figure S5). Interestingly, small matrix clumps were observed on day 3 and day 21, and the cell masses in the high–galactose concentration groups (35 and 70 mM) appeared partially sparse.

### 3.7 Cartilage-Related Gene Expression

Then, chondrocytes were assessed on days 3, 7, 14, and 21 for cartilage-related genes, such as type II collagen, aggrecan, type X, and type I collagen, to evaluate the influence on the chondrogenic phenotype. As depicted in Figure 3, the expression of the aggrecan gene (Figure 3B) was upregulated gradually during incubation and was significantly different from that of the control group on days 14 and 21. On day 14, there was a significant upregulation of aggrecan gene expression at galactose concentrations of 2 and 70 mM and a slight decrease at galactose concentrations of 8 and 35 mM. The aggrecan gene expression peaked on day 21, showing a significant increase with increasing galactose concentration. For type II collagen (Figure 3C), the gene expression levels were similar to those of aggrecan. Type II collagen gene expression levels were progressively upregulated with increasing galactose concentrations on days 3 and 7, while galactose concentrations resulted in increased expression at 2 and 70 mM. The type X collagen gene and type I collagen were also examined. There was a modest elevation in collagen type X (Figure 3D) and type I gene (Figure 3E) expression compared to that of the control group, which is likely to be a general phenomenon of drug treatment. In addition, the elevation of type II collagen and aggrecan was superior to that of type X and type I collagen after galactose treatment. In particular, for the type X collagen gene, there was no significant difference between the experimental groups before 14 days of culture, which indicated that galactose treatment may be more effective in promoting cartilage expression within 14 days.

### 3.8 Cartilage-Related Protein Expression *In Vitro*

**FIGURE 4:**
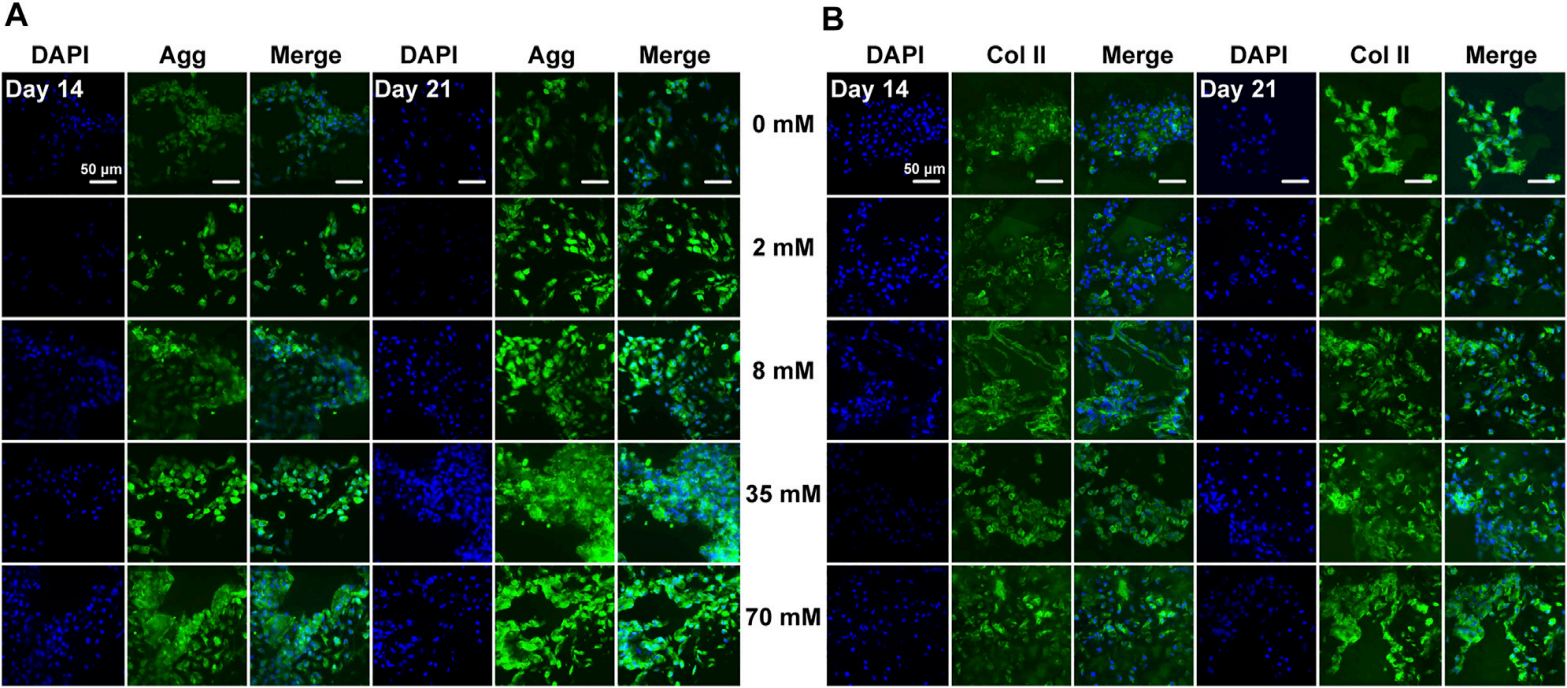
Immunofluorescence staining for the chondrogenic markers aggrecan **(A)** and type II collagen **(B)** of chondrocytes. Aggrecan and type II collagen were stained green, and nuclei were stained blue. Scale bar = 50 µm.

After the assessment of gene expression levels, immunofluorescence staining was performed under the same conditions to detect the protein levels of aggrecan, type II, type X, and type I collagen in chondrocytes. Consistent with the ATDC5 results, the protein expression was largely consistent with gene expression levels. For chondrocytes, staining was slightly stronger on day 21 than on day 14 and showed a significant increase in aggrecan (Figure 4A) and type II collagen (Figure 4B) expression with increasing galactose concentrations, demonstrating that galactose can successfully promote cartilage ECM secretion and accumulation. In addition, the protein expression levels of type X (Supplementary Figure S6A) and type I collagen (Supplementary Figure S6B) were visually different compared to those of the control group (0 mM). A moderate increase was observed in type I collagen levels at concentrations of 2 and 8 mM and was significantly weaker than that of aggrecan and type II collagen. Therefore, although there was an increase in the gene expression of type X and type I collagen in chondrocytes, associated with cell hypertrophy and fibrocartilage, no significant expression of type X and type I collagen was detected by immunofluorescence staining, suggesting that galactose also triggered anabolism and enhanced cartilage matrix formation by chondrocytes.

In particular, we also performed histological staining of the chondrocyte masses (H&E, safranin-O, and Masson) (Supplementary Figure S7). H&E staining was used to assess the overall structure of the *in vitro* cultured cartilage matrix, which showed that the chondrocytes were round and evenly distributed in the ECM. Safranin-O could detect the anionic GAG expression in the cartilage matrix. In Masson’s staining, the cytoplasm was stained red and the collagen fibers were stained blue. As the culture time was extended from 14 to 21 days, the matrix staining gradually deepened, indicating that the matrix secreted by the chondrocytes gradually increased. In addition, the formation of distinct cartilage lacunae in the cell masses was observed in the staining results, indicating successful cartilage matrix formation.

## 4 Discussion

OA, as a common joint disease worldwide, can lead to the erosion of joint cartilage and eventually the complete loss of movement function with severe pain that affects daily activities. Once OA, which is associated with mechanical injury, drugs, and aging, occurs, the cellular phenotype is altered, which results in a metabolic imbalance between the synthesis and degradation of the cartilage matrix, and the matrix gradually develops irreversible progressive lesions, and complicating inflammation (Bedingfield et al., 2021). However, the complex pathogenesis of OA, relevant cellular pathways, and therapeutic approaches have not been fully elucidated. Many methods of modifying the cell phenotype and promoting cartilage matrix regeneration are being investigated, and regulating cellular homeostasis is known to be a critical factor in altering the progression of and treating OA (Shi et al., 2019; Zhang et al., 2019; Ito et al., 2021; Sun et al., 2021).

Galactose, as an essential source of energy for people and carbohydrates for the cellular metabolism, is also an important component of GAGs, glycolipids, and glycoproteins (Cohn and Segal, 1973; Conte et al., 2021). Galactosylated glycoconjugates, such as glycoproteins, keratan sulfate–containing proteoglycans, and glycolipids, carry out a plethora of biological functions, including structural support, cellular adhesion, and intracellular signaling, while tissues such as the liver, kidney, and intestine require large quantities of UDP-galactose for the elaboration of GAG glycocalyx. The regulation of galactose metabolism in higher organisms has been studied from genetic, developmental, and enzymatic perspectives over the years. Due to its chemical structure, solubility, and dedicated transport system that allow diffusion across membranes, galactose has been increasingly studied in therapeutic applications (Yuan et al., 2018).

**SCHEME 2:**
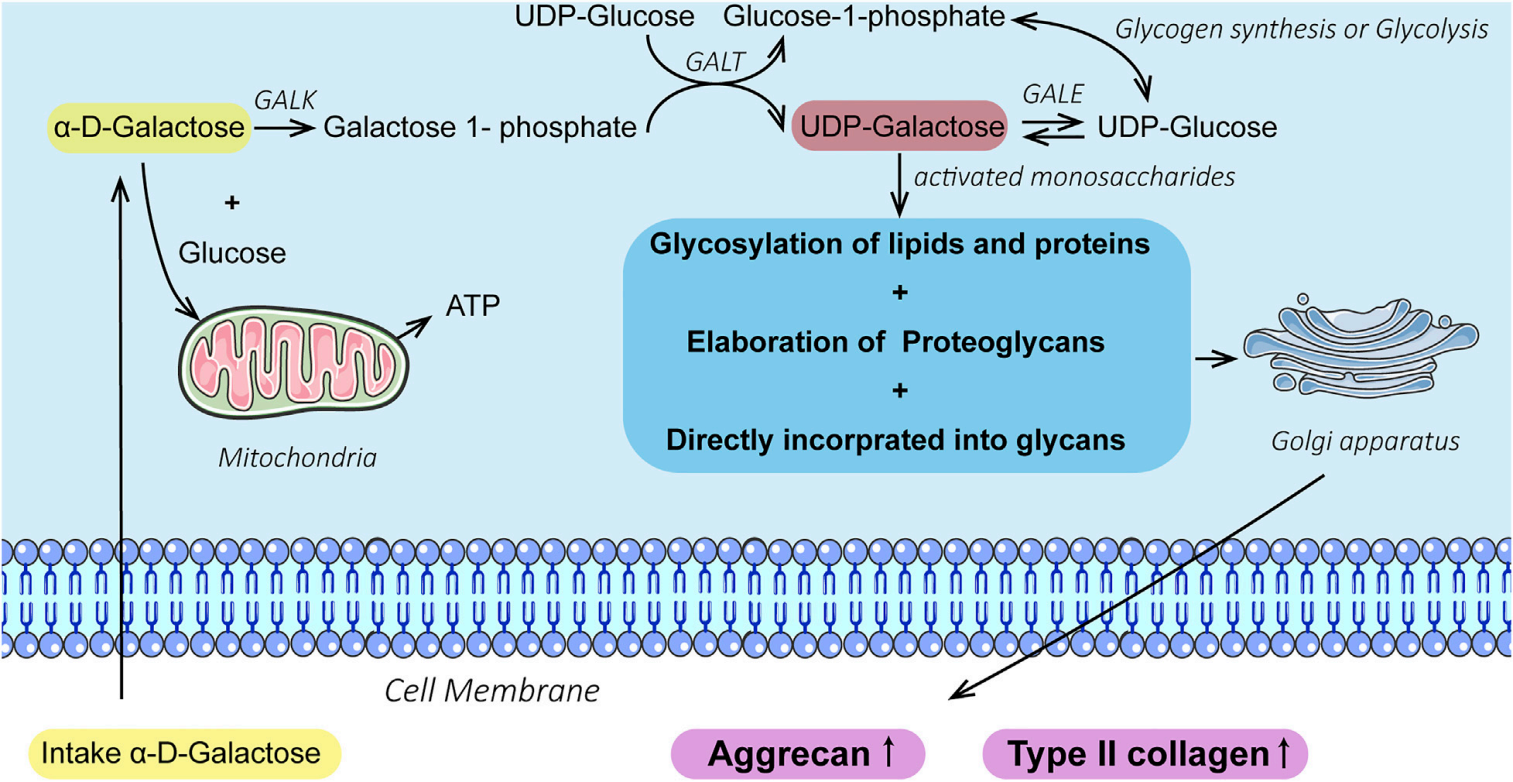
Illustration of galactose promoting chondrogenesis and ECM formation *via* the Leloir pathway catalyzed by the enzymes: galactokinase (GALK), galactose-1-phosphate uridyl transferase (GALT), and UDP-galactose 4-epimerase (GALE).

Herein, our results showed that galactose exerted positive effects on chondrogenic differentiation of ATDC5 and the synthesis of hyaline cartilage ECM in *in vitro* cell models of chondrocytes. First, supplementation with galactose did not significantly reduce cell viability in short-term culture (Supplementary Figures S1, S4), which is consistent with previous reports (Lane et al., 2015; Ohashi et al., 2021). The ability to metabolize galactose varies from cell to cell, and some cells are unable to metabolize galactose. Chondrocytes are highly specialized cells of mesenchymal origin that are responsible for producing, sustaining, and degrading cartilage ECM, which could lead to better adaptation to high galactose concentrations than ATDC5 cells, a cell line isolated from mouse teratocarcinoma fibroblastic cells (Gosset et al., 2008; Yao and Wang, 2013). Second, in our study, we observed that exogenously supplied galactose significantly increased the cell synthesis and secretion of GAGs (Figures 1A, 3A). Moreover, galactose significantly improved aggrecan and type II collagen expression both at the mRNA (Figures 1B,C, 3B,C) and protein levels (Figures 2, 4). The functions of GAGs are not limited to cell hydration and structural scaffolding, they also play a key role in cell signaling and regulating various biochemical processes (Raman et al., 2005). Humans can efficiently process large amounts of orally or intravenously administered galactose *via* the sugar nucleotide pathway, as shown by the rapid elimination of galactose from blood and the oxidation of ^14^C-galactose to ^14^CO_2_ (Segal and Cuatrecasas, 1968). The synthesis of proteoglycans by chondrocytes and osteoblasts was also observed in rats injected intraperitoneally with ^3^H-galactose within 24 h (Igarashi et al., 2013). This finding is due to galactose conversion into UDP-galactose, which is mediated by a series of four enzymes in the soluble fraction of the cell (Scheme 2), followed by direct incorporation directly into mature, *ab initio* glycans. This molecule is also involved in a variety of pathways, regulating cellular phenotypes and metabolic pathways (Radenkovic et al., 2019). Through this highly conserved Leloir pathway, galactose can be activated and act as a precursor for glycosylation or it can be converted to UDP-glucose, which then flows into glycogen synthesis or glycolysis, depending on the type of tissues and its energy requirements (Petry and Reichardt, 1998; Holden et al., 2003; Coelho et al., 2015; Christiansen-Weber et al., 2018). In our study, the accumulation of aggrecan and type II collagen (Figures 2, 4) was successfully visualized by immunofluorescence staining, explicitly mapping the outcome of the Leloir pathway.

On day 21, a significant increase in the type X collagen gene expression (Figures 1D, 3D) was observed at different concentrations, indicating cellular hypertrophy and cartilage calcification (He et al., 2014) possibly because galactose at high concentrations may result in cellular senescence, as previously reported (Han et al., 2021). In addition, the strong synthesis of GAGs in *in vitro* culture is often paralleled by the activation of the type X collagen gene and the expression of protein (Christiansen-Weber et al., 2018). However, galactose treatment was found to substantially reduce the production of nitric oxide and superoxide and downregulate hypoxia-inducible factor-2α expression, contributing to the anticatabolic effects, which alleviate the oxidative damage to cells by reactive oxygen species, thus maintaining redox homeostasis (Thoms et al., 2013; Lane et al., 2015). Replacing glucose in the medium with galactose was shown to promote mitochondrial respiration in chondrocytes and block downstream functional features associated with OA, including MMP13 and oxidation production (Ohashi et al., 2021). Thus, the external addition of galactose may be instrumental in activating anabolism and inhibiting catabolism.

In summary, galactose is critical to balancing all carbohydrate-based pathways in the presence of glucose and other sugars. Due to its properties, versatility, and the key role galactose plays in human metabolism, galactose and galactose-containing molecules have strong but untapped potential for nutritional, biotechnological, and pharmacological applications. In this study, galactose was characterized and demonstrated to be significantly less toxic than glucosamine reported in the literature and was able to significantly promote chondrogenic differentiation of the commonly used *in vitro* cell model ATDC5 and enhance the cartilage matrix formation by chondrocytes. The results of PCR and immunofluorescence staining showed that the effect of galactose was optimal within 8 mM. In conclusion, galactose and its modifications are worthy of continued research and exploration in the field of cartilage repair and drug delivery.

## Data Availability

The datasets presented in this study can be found in online repositories. The names of the repository/repositories and accession number(s) can be found in the article/Supplementary Material.
