# Supplementary material for: Galactose Enhances Chondrogenic Differentiation of ATDC5 and Cartilage Matrix Formation by Chondrocytes
Source: Front Mol Biosci. 2022 May 9;9:850778. doi: 10.3389/fmolb.2022.850778 (PMC9124793; doi:10.3389/fmolb.2022.850778)
Supplement: Supplementary file 1 [file DataSheet1.docx]

Supplementary Table 1. List of primers used in the RT-PCR analysis of gene expression in ATDC5 cells.

| Primer ID | Primers (5’- 3’) |
| --- | --- |
| GAPDH-F | TGTGTCCGTCGTGGATCTGA |
| GAPDH-R | TTGCTGTTGAAGTCGCAGGAG |
| Agg-F | AGTGGATCGGTCTGAATGACAGG |
| Agg-R | AGAAGTTGTCAGGCTGGTTTGGA |
| Col II-F | AGGGCAACAGCAGGTTCACATAC |
| Col II-R | TGTCCACACCAAATTCCTGTTCA |
| Col X-F | CTCCTACCACGTGCATGTGAA |
| Col X-R | ACTCCCTGAAGCCTGATCCA |
| Col I-F | ATGCCGCGACCTCAAGATG |
| Col I-R | TGAGGCACAGACGGCTGAGTA |

Supplementary Table 2. List of primers used in the RT-PCR analysis of gene expression in chondrocytes.

| Primer ID | Primers (5’- 3’) |
| --- | --- |
| GAPDH-F | CACCCACTCCTCTACCTTCG |
| GAPDH-R | GGTCTGGGATGGAAACTGTG |
| Agg-F | GGAGGTCGTGGTGAAAGGTG |
| Agg-R | CTCACCCTCCATCTCCTCTG |
| Col II-F | GTGGAAGAGCGGTGACTAC |
| Col II-R | TAGGTGATGTTCTGGGAGC |
| Col X-F | AGTTCTTCATTCCCTATGCCA |
| Col X-R | CAATGTCTCCTTTCGGTCCA |
| Col I-F | GGTGCTGCTGGTAAAGAAGG |
| Col I-R | GTCTACCCAAAGCACCAG |


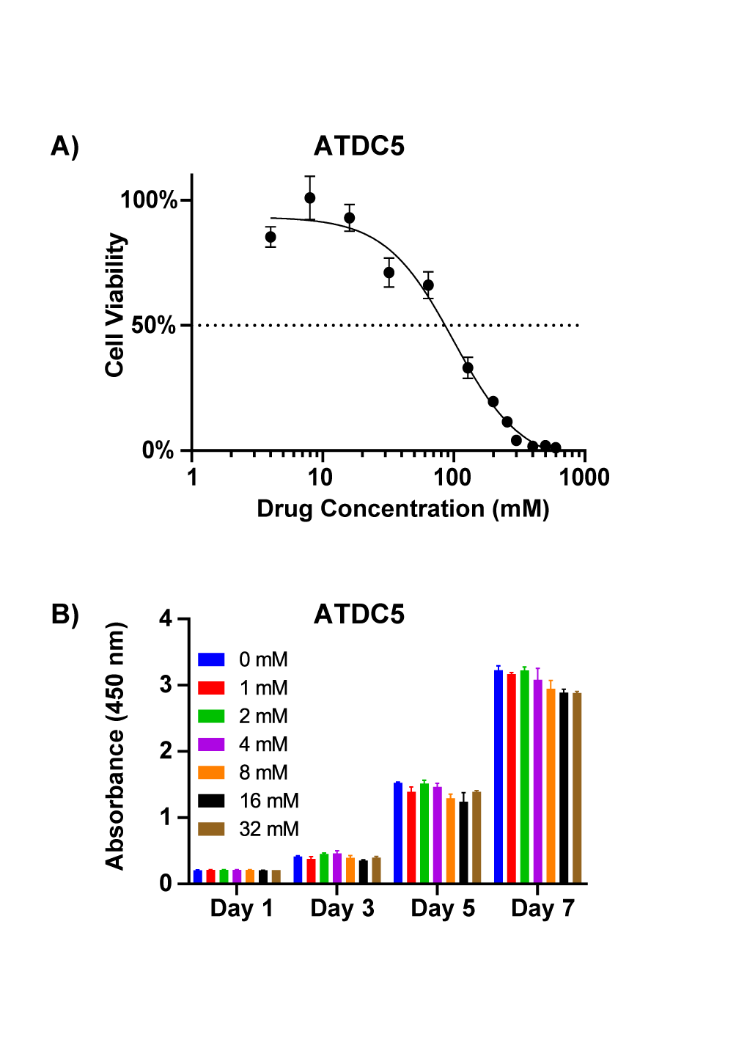


Supplementary Figure 1. Cell viability of ATDC5 cells when treated with different concentrations of galactose. (A) IC_50_ curves of ATDC5 towards galactose. Cells were cultured for 72 h. IC_50_ value of ATDC5 simulated by GraphPad is about 99.6 mM. (B) Proliferation histogram of ATDC5 co-cultured with a range of concentrations of galactose up to 7 days.


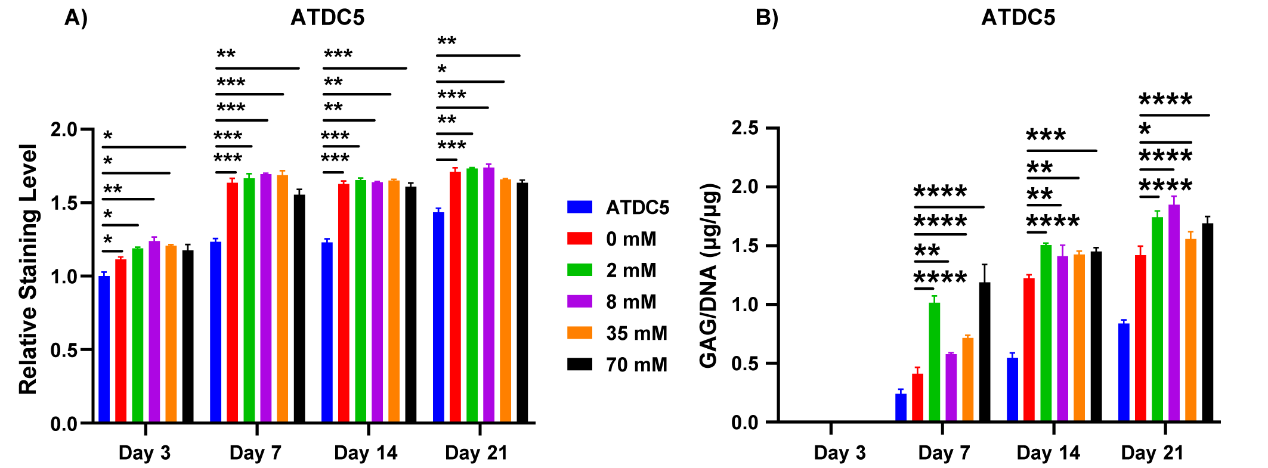


**Supplementary Figure 2**. (**A**) Quantitative analysis of Alcian blue staining of GAG accumulation in ATDC5 cells on day 3, 7, 14 and 21. (**B**) Quantitative analysis of GAG/DNA in ATDC5 cells, the amount of GAG was below detection limit on day 3.


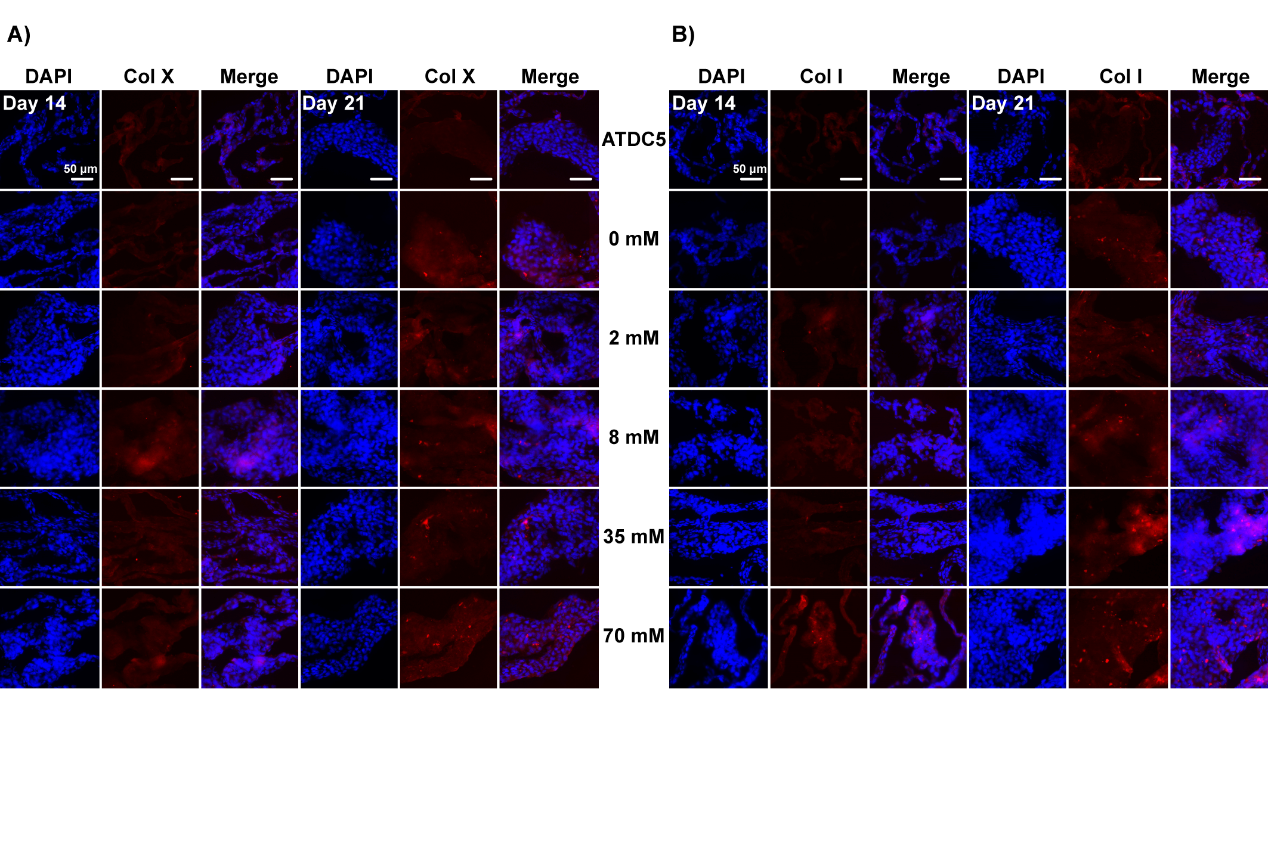


Supplementary Figure 3. Immunofluorescence staining for type X collagen (A) and type I collagen (B) of ATDC5. Type X collagen and type I collagen were stained with red and nuclei was stained with blue. Scale bar = 50 µm.


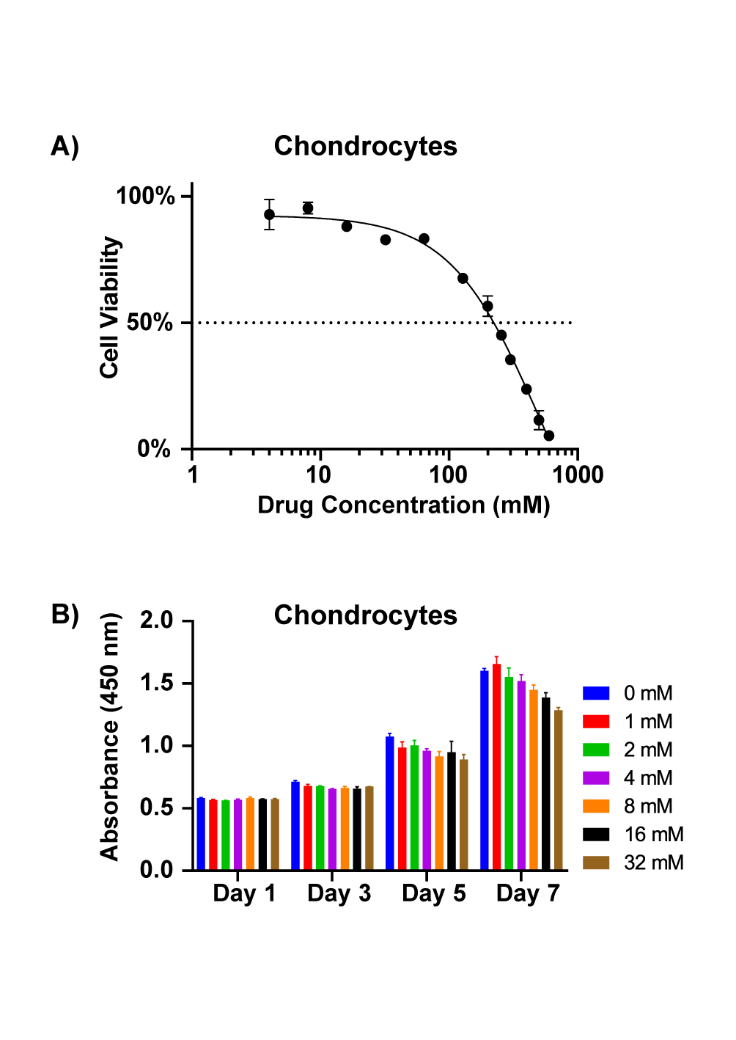


Supplementary Figure 4. Cell viability of chondrocytes when treated with different concentrations of galactose. (A) IC_50_ curves of chondrocytes towards galactose. Cells were cultured for 72 h. IC_50_ value of chondrocytes simulated by GraphPad is about 498.5 mM. (B) Proliferation histogram of chondrocytes co-cultured with a range of concentrations of galactose up to 7 days.


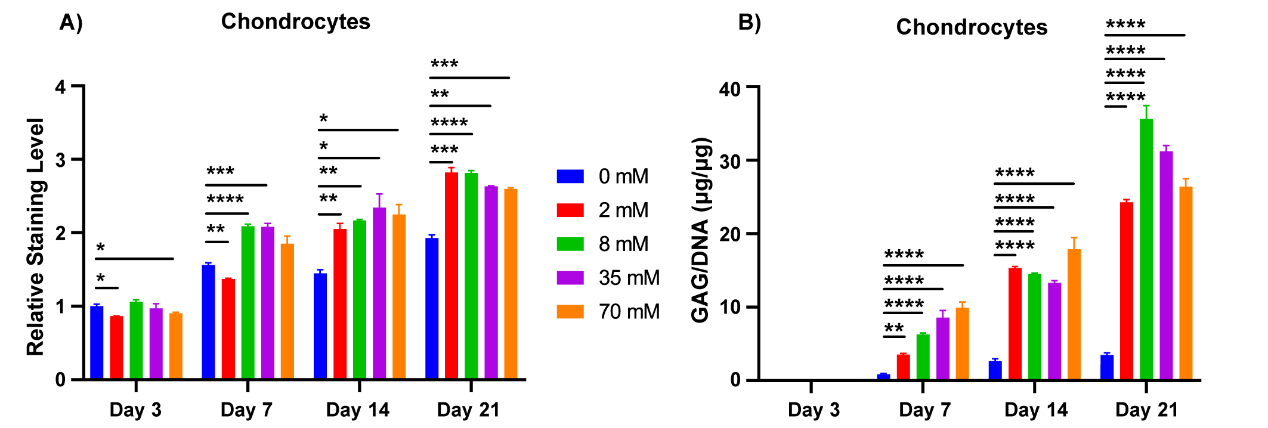


**Supplementary Figure 5**. (**A**) Quantitative analysis of Alcian blue staining of GAG accumulation in chondrocytes on day 3, 7, 14 and 21. (**B**) Quantitative analysis of GAG/DNA in chondrocytes, the amount of GAG was below detection limit on day 3.


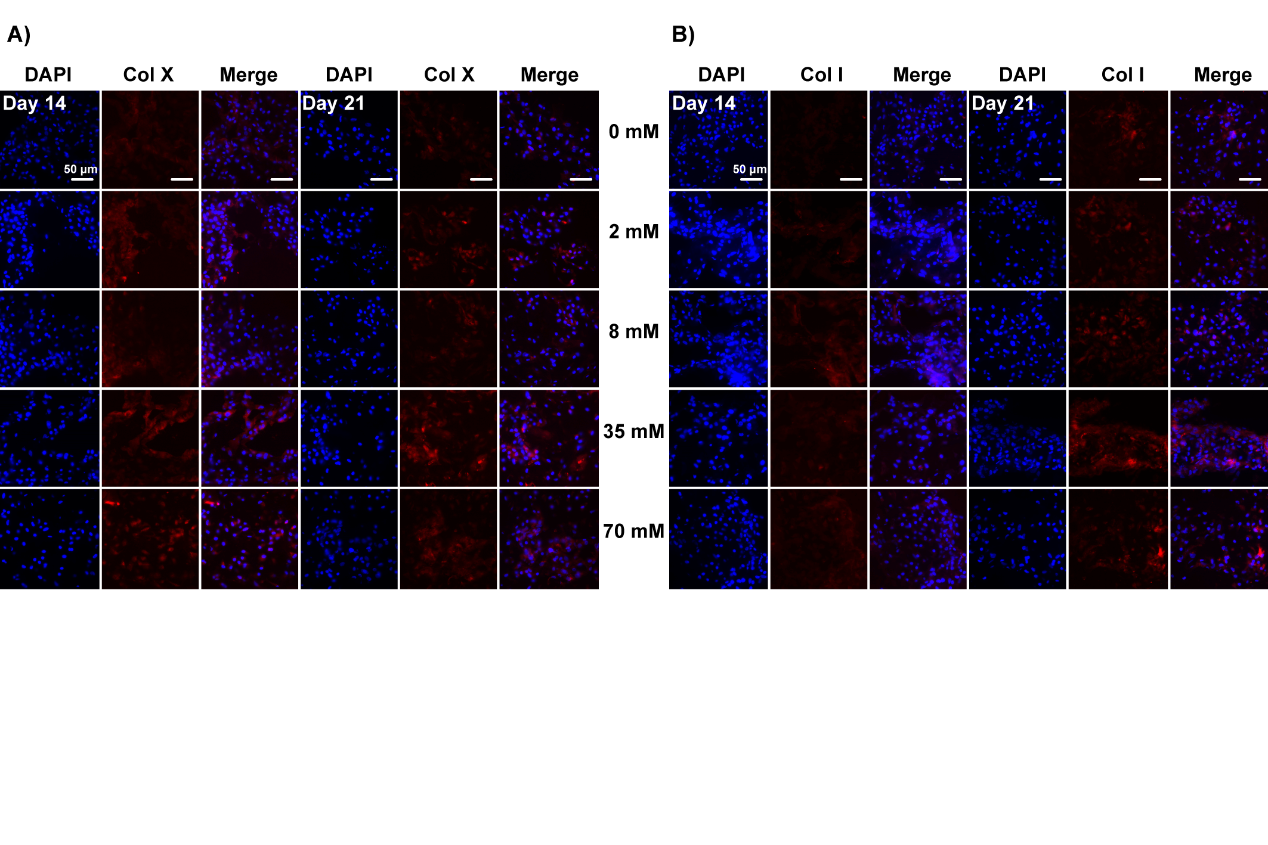


Supplementary Figure 6. Immunofluorescence staining for type X collagen (A) and type I collagen (B) of chondrocytes. Type X collagen and type I collagen were stained with red and nuclei was stained with blue. Scale bar = 50 µm.


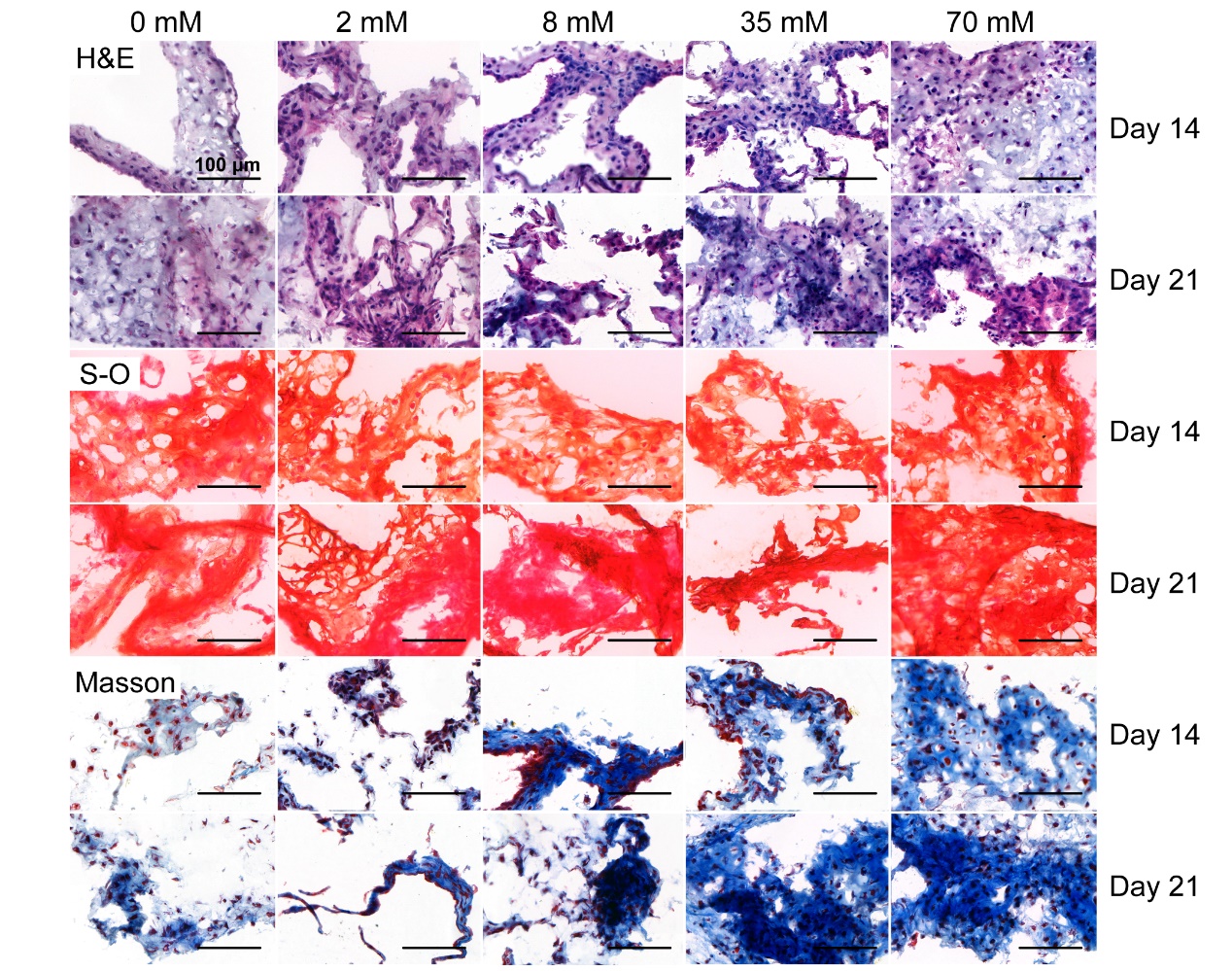


Supplementary Figure 7. Histological staining of chondrocyte masses (H&E, Safranin-O and Masson). Scale bar = 100 µm.
